# A comprehensive review on the implications of Yogic/*Sattvic* diet in reducing inflammation in type 2 diabetes

**DOI:** 10.1038/s41387-025-00371-0

**Published:** 2025-04-11

**Authors:** Anupama Vallazhath, Pooja Yedehalli Thimmappa, Harshit B. Joshi, Krishna Raghava Hebbar, Anupama Nayak, Shashikiran Umakanth, Apar Avinash Saoji, Nandi Krishnamurthy Manjunath, Basavaraj S. Hadapad, Manjunath B. Joshi

**Affiliations:** 1 Department of Ageing Research, Manipal School of Life Sciences, Manipal Academy of Higher Education, Manipal, 576104 India; 2 Division of Ayurveda, Centre for Integrative Medicine and Research, Manipal Academy of Higher Education, Manipal, 576104 India; 3 Department of Medicine, Dr. T.M.A. Pai Hospital, 576101 Udupi, India; 4 Swami Vivekananda Yoga Anusandhana Samsthana, Bangalore, 560105 Karnataka India; 5 Centre for Ayurveda Biology, Manipal School of Life Sciences, Manipal Academy of Higher Education, Manipal, 576104 India

**Keywords:** Type 2 diabetes, Obesity

## Abstract

Chronic inflammation in type 2 diabetes (T2D), characterized by constitutively activated immune cells and elevated pro-inflammatory mediators along with hyperglycaemia and increased free fatty acids and branched chain amino acid levels, significantly alters the immuno-metabolic axis. Over the years, dietary intervention has been explored as an effective strategy for managing T2D. Evidence from experimental and clinical studies indicates that various diets, including Mediterranean, Nordic, Palaeolithic and ketogenic diets, increase insulin sensitivity, decrease gluconeogenesis, and adiposity, and exert anti-inflammatory effects, thus preserving immuno-metabolic homeostasis in individuals with T2D. Indian dietary sources are categorized as *Sattvic*, *Rajasic*, and *Tamasic*, depending on their impact on health and behaviour. The Yogic diet, commonly recommended during yoga practice, is predominantly *Sattvic*, emphasizing plant-based whole foods while limiting processed and high-glycaemic-index items. Yogic diet is also recommended for *Mitahara*, emphasizing mindful eating, which is attributed to calorie restriction. Adopting a Yogic diet, featuring low-fat vegetarian principles, strongly reduces inflammatory mediator levels. This diet not only ameliorates insulin resistance and maintains a healthy body weight but also regulates immunomodulation, enhances gut microbiome diversity and provides essential phytonutrients, collectively preventing inflammation. Although, preliminary studies show aforementioned beneficial role of Yogic diet in improving diabetes associated metabolic and inflammatory changes, precise cellular and molecular mechanisms are not yet understood. Hence, further studies are warranted to decipher the mechanisms. This review summarizes the multiple roles of Yogic diet and related dietary components in mitigating inflammation and enhancing glycaemic control in T2D.

## Introduction

Type 2 diabetes (T2D) is associated with chronic and low-grade inflammation characterized by elevated levels of inflammatory mediators and constitutively activated immune cells. In response to various extrinsic and intrinsic factors, such as a sedentary lifestyle, obesity, hyperglycaemia, oxidative and ER stress and genetic and epigenetic reprogramming, the activation of inflammatory signalling pathways, such as the Jun N-terminal kinase (JNK) and nuclear factor-κ B (NF-κB) pathways, induces the constitutive production of inflammatory mediators. Studies have shown that inflammatory cytokines such as interleukin (IL)-1β, tumour necrosis factor-*α* (TNF-*α*), interleukin-6 (IL-6), MCP-1 and many others regulate metabolic processes under both physiological and pathological conditions. Chronic activation of the innate immune system associated with hyperglycaemia leads to persistent low-grade inflammation, which contributes to a range of complications, such as impaired β cell function, insulin resistance and impaired glucose homeostasis, leading to the onset and progression of diabetes and its complications [1–4]. Diabetes and obesity are metabolic disorders that manifest as a consequence of multiple factors, including the consumption of high-calorie foods, insufficient physical activity and a genetic predisposition. Excess consumption of processed grains, added sugars, fried foods, and processed and red meat elevates the risk of T2D-related diseases. Hence, the conventional approach to managing T2D involves medication, lifestyle modifications, and dietary interventions.

Various components in our diet induce both pro-inflammatory and anti-inflammatory signals, influencing immune responses within the gut. Different dietary nutrients directly interact with components of both innate and adaptive immunity or exert their effects by influencing the gut microbiota and its metabolites, thereby contributing to immune responses. In mouse models, a high-fat diet (HFD) caused an increase in the ratio of Firmicutes to Bacteroidetes, triggering the release of pro-inflammatory cytokines such as IL-6, IFN-γ, IL-1β and TNF-α resulting in signs of endotoxemia. A comparison study between whole and refined grain revealed that whole grain led to a reduction in energy intake, body weight, and low-grade systemic inflammatory mediators such as CRP and IL-6. Importantly, these noticeable changes were independent of the composition of the gut microbiome, underscoring the direct immune-modulating effects of whole grains [5]. Taken together, mounting evidence indicates that dietary factors regulate inflammatory pathways; hence, a tailored diet may help reduce inflammation in individuals with T2D and maintain metabolic homeostasis. Over the years, multiple dietary patterns, including the Mediterranean diet, Chinese diet and Palaeolithic diet have been examined to discern their influence on inflammatory processes in T2D. The essence of the Mediterranean diet philosophy involves prioritizing the consumption of legumes, vegetables, fruits, nuts, wholegrain foods, and fish. The results of several studies revealed that the Mediterranean diet significantly improves HbA1c levels, with greater adherence linked to a notable 23% reduction in the risk of developing T2D [6–8]. Compared to a diet designed for diabetes management, the Palaeolithic diet, also known as the caveman diet, significantly decreased the average HbA1c, triglyceride, diastolic blood pressure, weight, body mass index, and waist circumference [9].

The Yogic/*Sattvic* diet is an integral part of traditional yoga practices and has emerged as a potential complementary strategy in T2D management. This diet is often recommended during regular yoga, where the dietary pattern emphasizes the consumption of plant-based, whole foods while reducing the consumption of processed and high-glycaemic-index foods [10]. Despite the promising potential of the yogic diet as an adjunctive strategy in the holistic management and reduction of inflammation in T2D patients, further research is essential to elucidate the underlying precise mechanisms of action and long-term effects. This review aims to deepen our comprehension of the fundamental principles of the yogic diet and assess its effectiveness in managing glycaemic levels and reducing inflammation in individuals with T2D.

## Multiple pathways in T2D induce chronic low-grade inflammation in different tissues

Experimental and clinical studies have demonstrated the activation of tissue-specific inflammatory pathways and the induction of metabolic alterations affecting insulin sensitivity, gluconeogenesis, adiposity and the immune system. Adipose tissue inflammation plays an important role in regulating insulin sensitivity and glucose tolerance. During low-grade chronic adipose tissue inflammation, macrophages and other immune cell populations infiltrate the adipose tissue. An increase in the production of pro-inflammatory chemokines and cytokines such as C-C motif chemokine ligand 2, TNF-α, IL-1β and IL-6, as well as a decrease in the expression of the key insulin-sensitizing adipokine, adiponectin, is associated with the infiltration of pro-inflammatory cells into adipose tissue. Compared to subcutaneous fat, visceral fat has been shown to have a stronger association with insulin resistance [11]. The insulin receptor substrate-1 (IRS-1)/PI3K/Akt signalling axis is disrupted, contributing to reduced glucose transport and leading to hyperglycaemia [12]. Along with the increased secretion of proinflammatory cytokines (TNF-α and IL-6), adipose tissue macrophage infiltration and activation contribute to insulin resistance and impaired insulin signalling. The Toll-like receptor (TLR) pathway and the NOD-like receptor family pyrin domain containing 3 (NLRP3) inflammasome also play roles in connecting inflammation to metabolic dysfunction [2]. In rodent models of obesity-induced diabetes, macrophage infiltration is increased in pancreatic islets with a more pro-inflammatory phenotype [13].

Excessive lipid accumulation in non-adipose organs such as the liver and skeletal muscle leads to lipotoxicity and tissue damage. Ceramides and the intracellular lipid metabolite diacylglycerol (DAG) both activate the protein phosphatase 2 A (PP2A) and protein kinase C (PKC) pathways, affecting insulin signalling and reducing glucose absorption. Additionally, fatty metabolites may cause the unfolded protein response (UPR), increase ER stress and subsequently induce insulin resistance [14]. Emerging evidence indicates that T2D and obesity alter the gut microbiota composition, characterized by a reduction in beneficial bacteria and an increase in potentially pathogenic species [15]. Dysbiosis in the gut microbiota promotes inflammation, insulin resistance and metabolic dysfunction through multiple pathways, including the production of short-chain fatty acids (SCFAs) and endotoxins such as lipopolysaccharides (LPS). SCFAs influence insulin sensitivity and gluconeogenesis, while increased LPS levels contribute to systemic inflammation and insulin resistance [16].

Hyperglycaemia activates the NF-κB and JNK pathways, leading to pro-inflammatory milleu, impaired insulin signalling and insulin resistance in several insulin-responsive tissues, such as hepatocytes, skeletal muscles, adipocytes and endothelial cells [17, 18]. Compared to that in nonobese individuals, the expression of genes associated with inflammation in obese individuals was markedly elevated in abdominal subcutaneous adipocytes. The accumulation of lipids in adipocytes causes nicotinamide adenine dinucleotide phosphate (NADPH) oxidase activation, which exacerbates ROS production. Inflammation in T2D is primarily triggered by cytokines and adipokines released by adipocytes, including IL-6, TNF-α, resistin, visfatin, angiotensin, adiponectin, leptin, monocyte chemoattractant protein-1 (MCP-1), and plasminogen activator inhibitor-1 (PAI-1). Some of these proteins are also released by immune cells that are recruited to adipose tissue and participate in a feed forward loop for inflammation [19]. The major adipokines originating from adipose tissue during T2D are adiponectin and leptin. Among these, leptin shows significant pro-inflammatory activity [20]. PI3K signalling is required for leptin effects in the hypothalamus, and a dysfunctional pathway contributes to leptin resistance during diet-induced obesity. Leptin also triggers immune responses and induces other inflammatory mediators [21]. Increased amounts of IL-6 increase free-flowing fatty (FFA) synthesis and help inhibit insulin pathway signalling, which reduces the responsiveness of insulin in the liver and muscles. IL-6 regulates lipid metabolism, including suppressing triglyceride deposition and lipoprotein lipase (LPL) activity [22]. TNF-α attenuates the insulin signalling pathway by inhibiting insulin receptor tyrosine kinase activity in adipocytes and decreasing tyrosine phosphorylation and IRS-1 activation, which in turn reduces the ability of cells to respond to insulin. TNF-α has also been shown to decrease the expression of genes involved in insulin signalling [23]. It has been demonstrated that TNF-α stimulates the synthesis of chemokine (C-X-C motif) ligand 5 (CXCL5), a potent macrophage chemoattractant. Animals treated with anti-CXCL5 or those with C-X-C motif chemokine receptor 2 (CXCR2) knockouts exhibited reduced insulin resistance [24]. Resistin is a polypeptide that is crucial for numerous biological processes, including lipid metabolism, inflammation, and insulin resistance, as studied in rodent models. Recombinant human resistin causes insulin resistance via both the AMPK (AMP-activated protein kinase)-dependent and AMPK-independent suppressor of cytokine signalling-3 (SOCS-3) signalling pathways [25]. Data indicate that adiponectin regulates microRNAs to reduce intracellular proinflammatory pathways such as TLR-4 signalling, which contributes to some of the anti-inflammatory effects it has on adipose tissue [26]. IL-10 blocks the synthesis of pro-inflammatory cytokines, including TNF-α and IL-6, and exerts a key anti-inflammatory effect. Reduced amounts of IL-10, which is produced by lymphocytes and macrophages, are associated with metabolic syndrome and inflammatory responses in individuals with T2D via the reduction of tyrosine kinase activity of insulin receptors [27]. Nucleotide-binding oligomerization domain-like receptor 3 (NLRP3) is an inflammasome that functions in the production of IL-1β and IL-18, resulting in insulin resistance. In obese subjects with T2D, caloric restriction and exercise-mediated weight loss are linked to decreased NLRP3 expression in adipose tissue, as well as reduced inflammation and increased insulin sensitivity [28].

Adipocytes generate MCP-1 (CCL2), a chemoattractant for monocytes, dendritic cells, and memory T cells [29]. Weisberg et al., in 2006 demonstrated that knocking out C-C chemokine receptor type 2 (CCR2), a crucial MCP-1 receptor, shows decreased adipose tissue macrophage recruitment and inflammatory gene expression and further protected against insulin resistance in HFD models [30]. Glucose is the source of energy for immune cells, including neutrophils; hence, hyperglycaemia has an adverse impact on neutrophil function [31]. Previous studies have demonstrated that both diabetic and high glucose-pretreated neutrophils respond less strongly to stimuli such as LPS and form weaker and nonfunctional NETs [32]. In individuals with T2D, fasting and postprandial glucose levels are significantly correlated with neutrophil elastase and cell-free DNA [33]. It has been suggested that inflammatory pathways and excessive activation of T cells are also associated with the pathogenesis of T2D. The control mechanism regulating the generation of several sets of effector cytokines is closely associated with the differentiation of effector T cells [34]. T2D patients exhibit improperly differentiated T lymphocytes [35]. Although the proportion of B cells did not differ, the expression of CD38 (cluster of differentiation 38) in B cells was greater in normal individuals than in obese subjects with T2D [36]. Additionally, it was demonstrated that a HFD caused an increase in B-cell recruitment in adipose tissue [37].

Compared with those in nondiabetic donors, intra-islet increases in macrophages, along with polarization markers (CD11c, CD163, and NOS2) and proinflammatory cytokines (TNF-α, IL-6, and IL-1), have been demonstrated in T2D patients. IL-1β and IL-10 were expressed mostly by resident macrophages after clodronate-mediated depletion, whereas IL-6, TNF-α, and transforming growth factor β1 (TGFB1) were found to primarily originate from nonmacrophage sources in human islets [13]. TNF-α, IL-1β, and interferon-γ (INF-γ) have also been shown to contribute to β-cell malfunction and enhance susceptibility to β-cell toxicity. These cytokines increase the formation of the free radical nitric oxide (NO), which significantly slows cellular metabolism by impairing mitochondrial activity [38]. High FFA plasma levels are associated with glucose intolerance, disrupted muscle insulin signalling, increased hepatic gluconeogenesis, and reduced insulin response to glucose [39]. Additionally, the liver releases several acute-phase proteins in response to proinflammatory cytokines, including C-reactive protein (CRP), serum amyloid-A (SAA), alpha-1-acid glycoprotein (AGP), PAI-1 and haptoglobin [40]. The skeletal muscle is also considered a target of insulin resistance induced by inflammation [41]. Vascular cells also actively participate in inflammatory processes. The normal endothelium does not allow circulating leukocytes to adhere to it. However, under T2D conditions, the endothelium expresses cell adhesion molecules that adhere to leukocytes [42]. Our earlier studies showed that IL-6 induces proteasomal degradation of DNMT1 and induces DNA methylation-dependent gene expression related to insulin signalling and angiogenesis [43]. Taken together, the above studies demonstrate that the T2D microenvironment induces tissue-specific inflammation and that inflammatory mediators disrupt insulin signalling and consequently alter metabolic homeostasis.

## Dietary components modulate inflammatory and metabolic homeostasis

The influence of the immune system on nutrient distribution is apparent in various conditions, including food intake, obesity and exercise. Dietary intake plays a pivotal role in triggering cytokine activation and subsequent inflammation. Studies in animal models focusing on diet-induced obesity provide substantiating evidence that an organism’s diet profoundly affects the immune system. Studies have shown that obesity induces baseline elevation of proinflammatory cytokines such as IL-6 and TNF-α in the brain, with heightened levels in the cortex and hippocampus [44, 45]. The postprandial period is characterized by an intricate state involving endocrine, metabolic, and inflammatory processes. Studies have demonstrated that an elevated intake of dietary fat results in increased circulating levels of bacterial endotoxins such as LPS and proinflammatory cytokines in healthy individuals [46, 47].

Postprandial elevation of serum LPS indicates that metabolic endotoxemia induced by dietary fat may underlie the frequently observed postprandial inflammatory response. Elevated inflammatory cytokines, including TNF-α, IL-6, IFN-α, IL-1β, IFN-γ, IL-10, IL-12, and MIP-1β, have been observed after consumption of a high-fat meal in both individuals with type 1 diabetes (T1D) and healthy individuals. Notably, in the postprandial state, concentrations of triglycerides were found to be correlated with interleukin-10 (IL-10) and interleukin-12 (IL-12) [48, 49]. Ingestion of a high-fat, high-carbohydrate or combined diet induces postprandial inflammatory responses in healthy subjects, marked by increased plasma lipopolysaccharides, ROS production, IL-6, TNF-α, Nf-κB and elevated leukocyte counts. Various gut hormones, including glucagon-like peptide-1 (GLP-1), bile acids, leptin, FGF19 and ghrelin, play roles in the postprandial period and may exert anti-inflammatory effects [47, 50]. Furthermore, the levels of hormones related to the hypothalamic‒pituitary‒adrenal (HPA) axis, such as adrenocorticotropic hormone and cortisol, increase postprandially, which inhibits the production of several cytokines [47]. Given that the gut microbiome and dietary elements, such as saturated fatty acids, initiate inflammatory pathways via TLR4 and Nf-κB, numerous studies have focused on examining the impact of nutrition and dietary patterns on chronic low-grade inflammation.

The consumption of vegetables and fruits was associated with a decrease in baseline IL-6 levels, while the consumption of whole grains was linked to reduced TNF-α levels. Among various Mediterranean diets, the Cretan diet has been suggested to be particularly beneficial, potentially owing to its high intake of fresh vegetables, fruits, legumes and cereals. Adhering to the Mediterranean diet revealed a reduction in inflammatory markers, including CRP, IL-6, and ICAM-1, emphasizing the potential positive effects on inflammation and endothelial function [51]. In a cohort of healthy individuals, studies revealed a pronounced correlation between elevated weekly grain intake, particularly exceeding the 75^th^ percentile, and heightened serum levels of circulating TNF-α, MCP-1 and IFN-γ. Similarly, an intricate association was identified between red meat consumption and a statistically significant increase in IL-8 and CRP and a reiterated increase in IL-8. Furthermore, subjects exhibiting a proclivity for increased fruit consumption demonstrated elevated concentrations of interferon gamma-induced protein-10 (IP-10), IL-8, and IFN-γ. Notably, increased levels of IL-8 were detected in individuals who tended to consume more sweets. Conversely, a significant reduction in CRP levels was observed in individuals with augmented intake of eggs, greens, or shelled fruits. Within this subgroup, a statistically significant decrease in IL-6 and IL-1β was also observed, emphasizing the nuanced impact of specific dietary preferences on modulating inflammatory markers among healthy individuals [52]. These data suggest that dietary patterns, especially those emphasizing plant-based and Mediterranean approaches, play a crucial role in modulating inflammation, with specific food choices demonstrating intricate associations with pro- and anti-inflammatory markers in both healthy individuals and those with T2D.

## Multiple types of dietary patterns in the management of T2D-associated metabolic dysregulation

The manifestation and heterogeneity of the prevalence of T2D are influenced by a combination of factors, including diverse ancestry, varied dietary patterns, and heterogeneous edible oil consumption across different agroclimatic conditions. Notably, studies underscore the substantial impact of dietary choices on T2D predisposition. A balanced diet containing vegetables, fruits, lean meat, fish, and whole grain cereals rich in fibre, vitamins, and minerals lowers the risk of chronic diseases such as cancer, diabetes, or cardiovascular disease [53, 54]. Micha et al. [55] demonstrated that dietary variables contribute significantly to fatalities from heart disease, stroke, and T2D. A meta-analysis in European populations highlighted the association between increased consumption of red meat, processed meat, french fries, and refined grains and an elevated risk of developing T2D [56]. Dietary habits alter intricate molecular pathways in T2D that affect glucose metabolism, insulin sensitivity and inflammation. Hence, understanding these mechanisms is essential for developing targeted dietary interventions to enhance glycaemic regulation and overall metabolic health in individuals with T2D. Multiple studies have shown that poor adherence to diet and exercise regimens is a significant barrier to the use of nonpharmacological therapies for diabetes [57]. Excessive protein intake, particularly from fatty meat, results in elevated gluconeogenesis and increased blood glucose levels in individuals with T2D [58]. The consumption of high levels of dietary fats, including saturated and trans fats, has the potential to activate inflammatory pathways, induce TLRs and subsequently induce insulin resistance. A greater intake of processed grains, added sugars, fried foods, and processed or red meats may increase the risk of developing T2D. In children, the consumption of sugar-sweetened beverages has been identified as one of the contributing factors to the risk of childhood obesity [59], potentially leading to future diabetes-related complications. Chronic sugar consumption induces hyperinsulinemia and disrupts insulin receptor signalling, resulting in reduced cellular responsiveness to insulin. Excessive fructose consumption stimulates liver lipogenesis, contributing to non-alcoholic fatty liver disease (NAFLD), a common comorbidity of T2D. Contrary to expectations, zero-calorie drinks or diet soda appear ineffective and do not reduce the likelihood of developing T2D [60, 61]. A high intake of white rice increased the risk of developing T2D in Japanese women [62]. Epidemiological studies have revealed a significant association between diet and the incidence of T2D, with urbanization playing a crucial role. Nonetheless, there is a gap in information regarding the substantial contribution of fat intake to obesity, which potentially acts as a precursor to the development of T2D, as opposed to the intake of other macronutrients. Opting to avoid fast foods, fatty meats, sugar-sweetened beverages, and untimely food consumption while incorporating a diet rich in beneficial foods such as fruits, vegetables, and healthy dairy products reduces the likelihood of developing T2D [63]. A study by Yang et al., 2024, demonstrated a negative correlation between the onset of T2D and adherence to a prudent diet, characterized by high intake of whole grains, fruits, fish, and vegetables. Conversely, diets that restrict wheat, dairy, and eggs, as well as meat-based and full-cream dairy diets, exhibited positive associations with the onset of T2D [64]. Experiments on high fat diet induced obese mice by Ding et al., 2018 indicate that the administration of resveratrol (RES), a polyphenol predominantly present in grapes and mulberries, enhances CCR2 expression in white adipose tissue, mitigates inflammation, and diminishes macrophage infiltration, thereby improving insulin signaling markers in both subcutaneous and visceral adipose tissue. This strategy aids in preserving glucose metabolic equilibrium in obese mice caused by a high-fat diet [65].

In parallel with contemporary scientific findings, a plant-based diet rich in fruits, vegetables, whole grains, and legumes has demonstrated efficacy in improving lipid profiles, insulin sensitivity and glycaemic management in individuals with T2D [66]. In the context of T2D, the management of weight and glycaemic indices is effectively addressed through the practice of structured mindful eating [67]. Essential strategies for regulating blood glucose levels include maintaining portion control and distributing meals evenly throughout the day. The adoption of smaller, more frequent meals can mitigate sharp fluctuations in glucose levels, contributing to a more stable glycaemic profile [68]. A dietary focus on foods with a lower glycaemic index, coupled with an increased emphasis on complex carbohydrates such as whole grains and high-fibre foods, results in a decreased rate of glucose absorption. This dietary approach mitigates post meal spikes and augments glycaemic control [69]. Furthermore, the incorporation of unsaturated fats, particularly omega-3 fatty acids derived from sources such as nuts and avocados, exhibits anti-inflammatory properties and enhances insulin sensitivity in individuals with T2D [70]. The inclusion of soluble fibres in the diet contributes to the deceleration of glucose absorption, which is crucial for postprandial glucose management and overall glucose homeostasis [71, 72]. Notably, dietary fibres modulate the gut microbiota, positively influencing metabolic health and reducing inflammation. Short-chain fatty acids (SCFAs), which are produced in the gut by specific microorganisms, possess anti-inflammatory properties and may augment insulin sensitivity [73]. Epidemiological studies on T2D underscore that a greater consumption of fruits, vegetables, whole grains, and low-fat dairy products may diminish the risk of developing diabetes [74]. Studies have demonstrated a positive association between the consumption of vegetables, fruits, legumes, and dairy products and improved insulin activity, considering the glycaemic index and insulin activity alongside dietary habits [75]. Hence, cultivating a harmonious and healthy dietary regimen, coupled with consistent engagement in physical and mental exercise, may pave the way for a lifestyle devoid of disease.

In the context of T2D, caloric restriction alleviates oxidative stress and inflammation, as does autophagy. Autophagy restores pancreatic β-cell function and enhances glucose uptake by target tissues, including the liver and skeletal muscle [76]. Moreover, calorie restriction induces autophagy in liver cells, promoting hepatic insulin sensitivity and mitigating fatty liver disease, a prevalent consequence of T2D [77]. Notably, a deficiency in autophagy proteins exacerbates the expression of proinflammatory markers, indicating the intricate link between caloric restriction, autophagy, and the management of T2D [78].

### Classification of Indian dietary patterns

According to the *Triguna* hypothesis, physical makeup and mental attitudes are significantly regulated by regular dietary patterns [79]. The food we eat help us decides among consciousness, inertia, and agitation. Ancient yogic science divided food into three primary *categories Sattvic* foods, *Rajasic* foods, and *Tamasic* foods—based on how these food components affect the *trigunas* of the mind-body complex [80]. Yogic diet mainly encompasses a *sattvic* diet that includes a predominantly vegetarian diet, eaten with mindfulness and gratitude [81]. The food components classified under the *sattvic* category are fresh, nutrient-rich, naturally tasty, and light in the stomach, which are mostly consumed by those who practice yoga and those who aim for physio-psychological benefits. Eating such a diet increases life expectancy, inner and exterior strength, happiness, wisdom, health and satisfaction.

*Sattvic* foods encompass water, cereals, legumes, grains, fruits, most vegetables, nuts, and unrefined dairy products, such as unpasteurized and homogenized fresh milk, ghee, butter, paneer, cream, yogurt, and raw honey. Among these components, fresh milk from a contented cow and fruits fallen directly from trees are considered the purest manifestations of *sattvic* food, being unadulterated and perceived as a gracious gift from nature. Consuming a meal long after it has been prepared is not deemed *sattvic*, as the food is believed to have diminished in its inherent aura or energy [82, 83]. Food falling under the *rajasic* category is characterized by attributes such as spiciness, sourness, excessive sweetness or dryness. Consequently, individuals who partake in such foods are often characterized by intense passion and lead an active lifestyle. An appetite resembling that of a king aligns with the characteristics of a *rajasic* diet. However, the consumption of these foods is associated with the development of diseases, suffering, melancholy and restlessness attributed to the activation of *vata* and *pitta* in the body [84]. *Rajasic* foods include curd, nonvegetarian components; vegetables, including onion and garlic; and spices, such as pepper and chilies, along with lentils and pulses [85].

Food categorized as *tamasic* consists of food that has been stored overnight, leftovers, or has become stale. *Tamasic* foods include nonvegetarians, fermented foods, bread, cakes, alcohol, meat, and underripe and overripe vegetables and fruits [86]. It also encompasses overcooked, unclean, tasteless and rotten food, which tends to undergo microbial action, resulting in a loss of nutritional value. The consequences of consuming such a diet include feelings of laziness, lethargy, heaviness, irritability and doubt. Additionally, a *tamasic* diet contributes to accelerated aging and heightened drowsiness [87]. The ingestion of meat contributes to elevated levels of saturated fats and cholesterol present in a carnivorous diet, fostering the generation of bile acids. This process, followed by the conversion of these bile acids into deoxycholic acid and lithocholic acid by the gut microbiota, can lead to a reduction in microbial diversity and the occurrence of gut dysbiosis. These transformed acids can be toxic to beneficial gut bacteria [88]. Embracing a lifestyle rooted in yoga may enhance digestion, reduce inflammation, and contribute to weight loss, among various other health benefits [89].

### Components of yogic diet

A yogic or *sattvic* diet is generally fresh and light. Such dietary practices provide mental peace and clarity along with having a balanced, easily digestible diet that focuses on a healthy immune system. The yogic diet encompasses a low-fat vegetarian diet that ameliorates insulin resistance, helps maintain a healthy body weight, regulates immunomodulation, improves gut microbiome diversity, improves the gut microbiota, provides phytonutrients and prevents inflammation [66, 90, 91]. Adopting a yogic lifestyle, including yogic practices and a corresponding diet, has been shown to alleviate stress, anxiety and depression while also curbing food cravings and promoting a balanced body mass index.

According to Ayurvedic concepts, adherence to a balanced *Sattvic* diet is purported to enhance vitality, strength, and vigor [54]. In the yogic diet, *Mitahara* encompasses the concepts of calorie restriction, mindful eating and consuming food that are only congenial to health and wellbeing. Calorie restriction in individuals with T2D contributes to weight loss, positively influencing insulin sensitivity and glycaemic control [92]. *The* practice of *Mitahara* leads to a reduction in visceral fat, a key contributor to inflammation, thereby decreasing proinflammatory cytokines and adipokines [93]. Traditional yoga scriptures, such as *Hatha Pradipika and Gheranda Samhitha*, provide invaluable guidance on dietary choices for Yoga Sadhakas (practitioners) to optimize their benefits [94]. The prescribed diet for yogic practitioners includes fresh whole lcereals, butter, fruits, and vegetables, in alignment with the principle of *Mitahara*, emphasizing easily digestible and mind-pleasing food choices. A yogic diet recommends nutrient-dense foods, and mindful eating aligns seamlessly with the overarching goals of T2D management. As a result, a burgeoning body of evidence suggests the potential benefits of integrating yoga and dietary modifications in the comprehensive approach to T2D management, underscoring the need for more extensive research to elucidate their multifaceted roles.

**Fig. 1:**
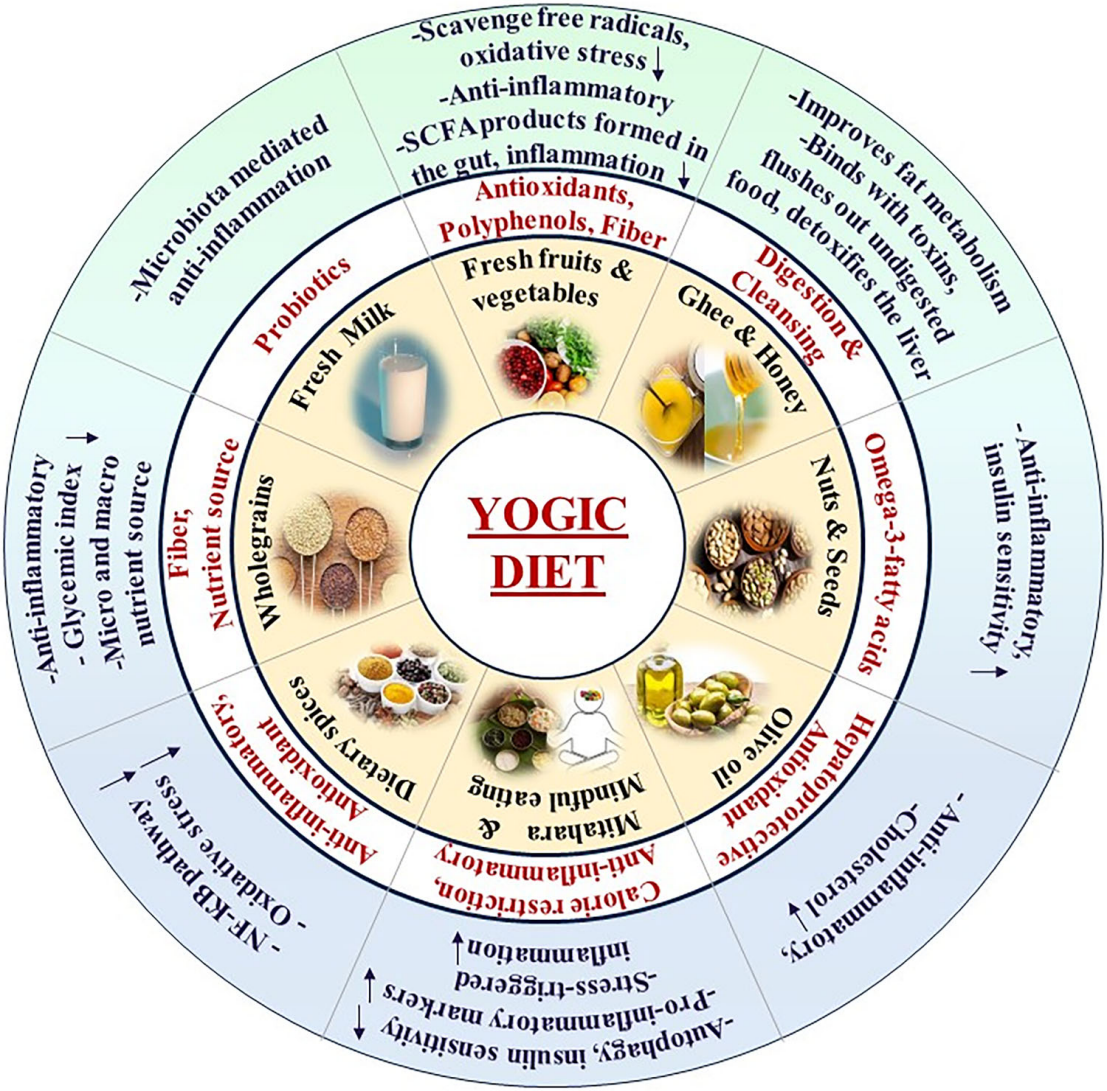
Yogic diet in health and diseases. The yogic diet encompasses a fresh, plant-based diet and unpasteurized dairy products rich in bioactive compounds, vitamins, minerals, probiotics, fibre, and healthy omega-3 fatty acids that reduce T2D-induced inflammation and ameliorate metabolic health.

Fresh fruits and vegetables are an integral part of the yogic diet and are rich sources of antioxidants that contain fibre and function in improving digestion [95]. Whole grains, rice and easily digestible pulses are incorporated because these foods contain micro- and macronutrients and are carriers of vitamin B. Two essential components that are recommended for daily consumption are honey and ghee. These factors help in easy digestion and overall cleansing [96]. The source of probiotics is fresh milk, which is an inevitable part of the yogic diet and contributes extensively to good health [97]. A yogic diet is generally considered pleasant and sweet in taste. However, moderate levels of sugar, which is pleasant to the mind and aids in digestion, must be consumed [82]. Nuts are strong sources of healthy omega-3 fatty acids [66, 95]. India is known for its spices, and anti-inflammatory dietary spices, such as cinnamon, turmeric, ginger, coriander, cardamom and saffron, are derived from plants used in Ayurveda. These compounds are extensively included in the yogic diet and have been shown to suppress inflammatory cytokines and NF-κB signalling pathways, which contribute to the pathogenesis of diseases such as cardiovascular diseases and T2D [98] (Fig. 1).

Spicy food, including chilly, onion and garlic, increases *tamas* and *rajas* in the body and reduces *agni* [10]. Dietary components, including curd, garlic, nonvegetarian food, and oils such as mustard and sesame, are considered to be stimulatory and fierce in passion due to their salty and pungent nature. Since yoga involves internal arousal of the nervous system, a yogic diet avoids foods that cause external stimulation [85, 94, 95]. Meat is avoided because it is highly fat and unecological and contains adrenaline, which can cause fear. Refined grains and pulses are not included in the Yogic diet due to loss of mineral content during refining and may cause constipation. In addition, it is essential to eat a yogic diet according to the agro-climatic region and at proper times. This is because the cycle of the body is closely connected to the natural habitat [10].

### Yogic diet ameliorates inflammation in T2D

Studies have shown that dietary modifications play a crucial role in mitigating inflammation associated with T2D [99, 100]. Some preliminary studies suggest that a yogic diet may play a beneficial role in improving diabetes-related metabolic and inflammatory changes. However, the precise cellular and molecular mechanisms remain unclear due to the diverse dietary patterns followed across various agroclimatic regions of the Indian subcontinent, which contribute to the vast heterogeneity and complexity in defining a sattvik or yogic diet. A study on diabetic postmenopausal women examined inflammatory mediators after a prescribed diet change and exercise regimen. The authors demonstrated that exercise alone did not cause much change, and there was a difference in the various inflammatory markers in response to a low-fat diet. Plasma CRP levels and leptin levels were decreased upon intervention with dietary modifications along with exercise, whereas few changes were found in adiponectin and TNF-α [101]. A yogic diet positively affects inflammatory markers and stress markers within 10 days of the regimen. The levels of these markers, including IL-6 and TNF-α, were significantly reduced [102]. A study in 72 subjects with obesity suggested that diet-based interventions had beneficial effects on aging and inflammatory processes. The major outcomes were the relative fold changes in the expression of genes related to oxidative stress, NF-κB, IL-6, TNF-α, and human telomerase reverse transcriptase (TERT) in peripheral blood mononuclear cells (PBMCs) [103].

According to Das et al. (2023), a yogic diet encourages the consumption of anti-inflammatory foods such as fruits, vegetables, whole grains, nuts and seeds [91]. These dietary components are full of bioactive substances such as vitamins, minerals, polyphenols, and antioxidants that have been found to control inflammatory pathways. Antioxidants scavenge free radicals and lessen oxidative stress, which is a significant factor in T2D-related inflammation [104]. Polyphenols inhibit inflammatory enzymes, such as cyclooxygenase (COX) and lipoxygenase (LOX), thus suppressing the production of pro-inflammatory mediators [105]. Plant-based diets are also associated with reduced cardiovascular risk factors in individuals suffering from T2D [66].

Ghrita, an ayurvedic formulation which constitutes ghee from cow milk, is considered to be the superior of fats and good for oleation. Ghee is rich in conjugated linoleic acid and proven to be antidiabetic. However, with ghee rich in linoleic acid, demonstrated a reduction in prostaglandins, leucotrines and interleukins associated with inflammation. In subjects with T2D or metabolic syndrome, consumption of milk and dairy products did not show pro-inflammatory (neutral) or showed an anti-inflammatory effect [106–108]. Numerous clinical studies have demonstrated that substituting honey for sucrose and dextrose results in lowered glucose levels, diminished postprandial glycaemia in healthy individuals, and a reduced postprandial glycaemic response [109–112]. The effect of honey on cell cultures have shown to reduce inflammatory mediators such as TNF- α, IL-1 β, IL-6 and also inhibit TLR4/NFκB expression [113]. Several studies have demonstrated the influence of natural honey consumption among healthy human subjects and has been reported to reduce post-prandial glycaemic response and glucose level in the blood [110, 111, 114, 115]. The study demonstrated that hesperidin, a flavanone glycoside present in citrus fruits, plays a role in glucose homeostasis. Consumption of hesperidin supplements may enhance glycaemic control through improved antioxidant effects. Hesperidin significantly influences glucose transporters (GLUTs) by down-regulating hepatic GLUT2 expression and up-regulating GLUT4 expression in adipocytes. Additionally, it has been reported that hesperidin enhances the expression of adipocyte PPAR genes and glucokinase [116]. Dairy products and dairy proteins have demonstrated efficacy in reducing inflammatory markers, including CRP, IL-6, TNF-α, and MCP-1, in individuals with obesity and overweight conditions [108, 117]. Whole grains have contributed to stable HbA1c levels, and lower concentrations of C-peptide and leptin in healthy subjects [118]. Furthermore, legume seeds, especially peanut butter, have demonstrated potential in decreasing inflammatory biomarkers, including total cholesterol, LDL, and apolipoprotein B (apo B), in women with T2D [119]. Furthermore, the prudent diet shown an inverse correlation with the majority of inflammatory markers, while the full-cream dairy diet showed a positive correlation with these markers. Significantly, the majority of inflammatory markers, especially the INFLA-score, exhibited a strong correlation with the onset of T2D. The INFLA-score mediated 13% of the link between the sensible diet and the incidence of T2D, and 34% of the association between the full-cream dairy diet and the beginning of T2D [64]. Linoleic acid (LA) (18:2n-6), an essential fatty acid that derived primarily from plant oils and legumes, constituted 85–90% of dietary n-6 PUFAs. According to a meta-analysis investigation of the dose-dependent relationship between LA and the risk of T2D demonstrated that greater intake of LA reduces risk T2D [120]. Flaxseed, an excellent source of omega fatty acids, has been shown to decrease serum glucose and insulin levels in overweight/obese individuals with pre-diabetes after 12 weeks of consumption [116, 121]. Studies indicates that the consumption of vegetables is connected with a 9% reduction in the incidence of T2D with an increased intake of up to 300 g/day, consumption of fruits up to 200–300 grams per day lowered T2D risk by 10 and dairy consumption diminished the risk of T2D by 6% with increased intake up to 400–600 g/day [122].

Plant-based proteins, such as those from legumes, nuts, and seeds have been shown to reduce inflammation, likely due to their anti-inflammatory properties and relatively high fibre content [123]. Dietary spices have played a major role in the Indian diet since the inception of Ayurveda and are known for their anti-inflammatory properties. It has been demonstrated that cinnamaldehyde and cinnamon treatment leads to downregulation of the NF-κB pathway and reduces the levels of proinflammatory cytokines in diseases such as T2D and atherosclerosis in ApoE^−/−^ mice [124, 125]. The focus of yogic diets on nutrient-dense, plant-based foods, along with the prevention of overeating, can aid in weight management, an important aspect of T2D management. Given that adipokines such as adiponectin and leptin are secreted by adipose tissue in obesity, which leads to inflammation, weight loss following a yogic diet and lifestyle changes lessens inflammation in adipose tissue and increases insulin sensitivity [11]. A comparative approach revealed that a low-carbohydrate diet was more beneficial than a low-fat diet (LFD) for a better inflammatory state, as indicated by the levels of interleukins and tumor necrosis factors in the serum [126].

**Fig. 2:**
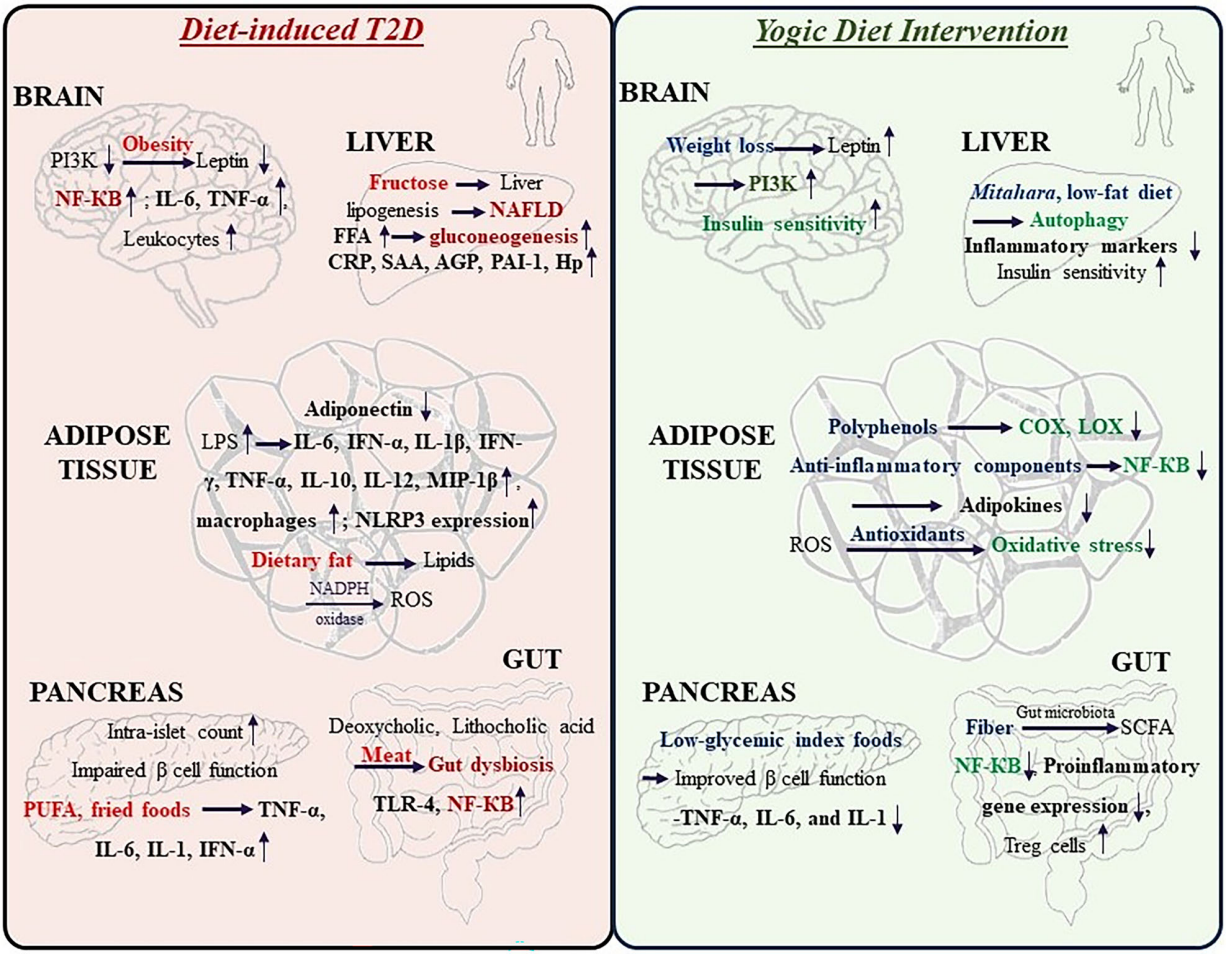
A yogic diet aids in reducing inflammation related to type 2 diabetes through various mechanisms and signalling pathways. Obesity due to diet-induced T2D leads to downregulation of the insulin signalling pathway followed by heightened levels of proinflammatory cytokines in the cortex and hippocampus. In the liver, excessive fructose ingestion leads to lipogenesis in the liver, which contributes to nonalcoholic fatty liver disease (NAFLD). Insulin sensitivity is reduced, gluconeogenesis is increased, and inflammation is increased. Adipose tissue is characterized by the infiltration of proinflammatory cytokines and immune cells as well as lipid-induced oxidative stress. In the pancreas, high amounts of polyunsaturated fatty acids (PUFAs) and fried foods lead to an increase in proinflammatory cytokines. Gut dysbiosis is a comorbidity of diet-induced T2D that leads to inflammation. Weight loss due to a yogic diet, including lifestyle, improves the insulin signalling pathway and insulin sensitivity in the target tissues. When a yogic diet is introduced, caloric restriction promotes autophagy in liver cells, improving tissue insulin sensitivity and reducing inflammation and NAFLD. Polyphenols and antioxidants derived from yogic diets reduce inflammation and oxidative stress. Recruiting low glycaemic index foods as a part of the yogic diet promotes improved β cell function and reduced proinflammatory cytokine secretion. Certain gut microbes metabolize dietary fibre into short-chain fatty acids (SCFAs), which inhibit inflammation and improve insulin sensitivity.

The emphasis of the yogic diet on fibre-rich foods supports gut health and promotes a diverse and balanced gut microbiota composition. A healthy gut microbiota is associated with reduced inflammation and improved metabolic health. Certain gut microbes metabolize dietary fibre into SCFAs, such as butyrate, acetate, and propionate, which possess anti-inflammatory properties [127]. SCFAs inhibit NF-κB activation, suppress proinflammatory gene expression, and promote the generation of regulatory T cells, which help maintain immune balance [128]. Mindful eating practices, an integral part of the yogic diet, reduce stress and promote relaxation [129]. Inflammation in T2D patients is worsened by chronic stress, which further leads to an increase in pro-inflammatory mediators [130]. Mindful eating habits, such as being present and grateful for each meal, can lower stress hormone levels and help alleviate inflammation. The yogic diet stresses whole, unprocessed foods and the avoidance of refined sugars and unhealthy fats that help improve insulin sensitivity. Upon promoting better glycaemic control and reducing hyperinsulinemia, the yogic diet may directly and indirectly contribute to the reduction in inflammatory pathways associated with insulin resistance (Fig. 2).

## Conclusion

The intricate interplay among chronic inflammation, lifestyle factors and T2D underscores the importance of holistic management. From the detrimental effects of poor dietary choices to the positive impacts of mindful eating, plant-based diets, and traditional practices such as Ayurveda and yoga, it is clear that nutrition plays a critical role in T2D development, management, and prevention. Conventional strategies involve medication, lifestyle modifications, and dietary interventions, and hence, the practice of the yogic diet as a complementary strategy offers a promising avenue. Studies highlight the beneficial impact of dietary patterns such as *sattvic* and Mediterranean diets on reducing inflammation, emphasizing the nuanced role of individual food groups in influencing inflammatory markers. Dietary modifications, particularly those promoting whole foods and avoiding inflammatory triggers, offer a potential strategy for alleviating inflammation in patients with T2D. Further research is essential to fully comprehend the cellular and molecular mechanisms and long-term impact of the yogic diet on T2D management.
